# Studying the Mechanism of *Plasmopara viticola* RxLR Effectors on Suppressing Plant Immunity

**DOI:** 10.3389/fmicb.2016.00709

**Published:** 2016-05-18

**Authors:** Jiang Xiang, Xinlong Li, Jiao Wu, Ling Yin, Yali Zhang, Jiang Lu

**Affiliations:** ^1^The Viticulture and Enology Program, College of Food Science and Nutritional Engineering, China Agricultural UniversityBeijing, China; ^2^Guangxi Crop Genetic Improvement and Biotechnology Laboratory, Guangxi Academy of Agricultural SciencesNanning, China

**Keywords:** *Plasmopara viticola*, grapevine, RxLR effector, cell death, immunity

## Abstract

The RxLR effector family, produced by oomycete pathogens, may manipulate host physiological and biochemical events inside host cells. A group of putative RxLR effectors from *Plasmopara viticola* have been recently identified by RNA-Seq analysis in our lab. However, their roles in pathogenesis are poorly understood. In this study, we attempted to characterize 23 PvRxLR effector candidates identified from a *P. viticola* isolate “ZJ-1-1.” During host infection stages, expression patterns of the effector genes were varied and could be categorized into four different groups. By using transient expression assays in *Nicotiana benthamiana*, we found that 17 of these effector candidates fully suppressed programmed cell death elicited by a range of cell death-inducing proteins, including BAX, INF1, PsCRN63, PsojNIP, PvRxLR16 and R3a/Avr3a. We also discovered that all these PvRxLRs could target the plant cell nucleus, except for PvRxLR55 that localized to the membrane. Furthermore, we identified a single effector, PvRxLR28, that showed the highest expression level at 6 hpi. Functional analysis revealed that PvRxLR28 could significantly enhance susceptibilities of grapevine and tobacco to pathogens. These results suggest that most *P. viticola* effectors tested in this study may act as broad suppressors of cell death to manipulate immunity in plant.

## Introduction

During co-evolution with microbial pathogens, plants have evolved multiple layers of innate immune surveillance that successful pathogens have evolved to evade or suppress. The first layer is comprised of pattern recognition receptors (PRRs) that recognize broadly conserved pathogen molecules (pathogen/microbe-associated molecular patterns, PAMP/MAMPs), and activate defense responses including the induction of defense genes, production of reactive oxygen species (ROS) and deposition of callose (Jones and Dangl, 2006). This recognition leads to the so-called PAMP (or pattern)- triggered immunity (PTI; Katagiri and Tsuda, 2010). However, successful pathogens can deliver effectors into plant cell to suppress or interfere with PTI, resulting in effector-triggered susceptibility. The second layer of defense is mediated by resistance (R) proteins that directly or indirectly recognize the presence of pathogen effectors, leading to effector-triggered immunity (ETI; Jones and Dangl, 2006). A hallmark of this recognition is the programmed cell death (PCD), termed hypersensitive response (HR) which helps resist biotrophic pathogens (Dodds and Rathjen, 2010). However, this distinction between PAMPs and effectors, or between PTI and ETI, is blurred and has been challenged by recent studies (Thomma et al., 2011). Additionally, not all microbial defense activators conform to the common distinction between PAMPs and effectors, and downstream defense pathways in plants activated during PTI and ETI often overlap and operate against a broad spectrum of pathogens. These layers of immunity likely function together as a continuum (Thomma et al., 2011).

Many plant pathogens can secrete effector proteins to counteract immune response of plant. The role of effector proteins in pathogenesis has been characterized most extensively in bacteria. For example, it has been well known that bacterial species could secrete 20–40 effectors into the cytoplasm of plant cells via a type-III secretion system (TTSS; Grant et al., 2006). During infection, many of these bacterial effectors suppress defense responses, PTI and/or ETI, through a variety of mechanisms (Hann et al., 2010). In contrast, researches on effectors from fungi and oomycetes are relatively few.

Oomycete pathogens are a phylogenetically distinct eukaryotic lineage within the Stramenopiles and are evolutionary related to brown algae, which cause some of the most destructive plant diseases and result in huge economic losses in the world (Soanes et al., 2007; Stassen and Van den Ackerveken, 2011). Over the last few years, the genomes of several hemibiotrophic oomycete pathogens, including *Phytophthora ramorum* (the causal agents of sudden oak death), *Phytophthora sojae* (soybean root rot pathogen) and *Phytophthora infestans* (potato late blight pathogen), have been sequenced (Tyler et al., 2006; Haas et al., 2009). The genomes of two biotrophic oomycete pathogens, *Hyaloperonospora arabidopsidis*, a pathogen causes downy mildew in *Arabidopsis thaliana* (Baxter et al., 2010), and *Plasmopara halstedii*, which infects sunflower germlings and young plantlets (Sharma et al., 2015), have also been sequenced. Bioinformatic analysis of the sequenced genomes revealed that oomycete pathogens maintain an extraordinarily large superfamily of secreted proteins that could potentially act as effectors. The superfamily of effectors can broadly be divided into two groups: apoplastic effectors and host-translocated effectors. Host- translocated effectors were mainly identified as Crinklers and RxLR effectors (Stassen and Van den Ackerveken, 2011). The most extensively studied effectors are RxLR effectors, defined by a conserved N-terminal secretory signal peptide followed by an RxLR amino acid motif. However, many aspects of the mechanisms by which oomycete RxLR effectors target to the interior of plant cells remain unsolved (Petre and Kamoun, 2014).

With 134 putative *RxLR* effector genes in *H. arabidopsidis*, 260 RxLR-like proteins in *P. halstedii*, over 350 predicted RxLR effectors in *P. ramorum* and *P. sojae*, and more than 550 *RxLR* sequences in the genome of *P. infestans*, assessing functions of all RxLR effector candidates is a tremendous challenge (Tyler et al., 2006; Haas et al., 2009; Baxter et al., 2010; Sharma et al., 2015). Thus, the development of a medium/ high- throughput system to explore their function in plants is strongly desired. Recently, large-scale function surveys to identify avirulence or virulence role of predicted RxLR effectors from oomycetes have already been undertaken. Until now, only a small number of avirulence genes have been characterized from *Phytophthora* species and *H. arabidopsidis*, including *P. sojae Avr1a* (Qutob et al., 2009), *Avr1b* (Shan et al., 2004), *Avr3a* (Dong et al., 2011b), *Avr3b* (Dong et al., 2011a), *Avr3c* (Dong et al., 2009), *Avr4/6* (Dou et al., 2010), *Avr5* (Dong et al., 2011b), *P. infestans Avr2* (Gilroy et al., 2011), *Avr3a* (Armstrong et al., 2005), *Avrblb1* (Vleeshouwers et al., 2008), *Avrblb2* (Oh et al., 2009), *Avr4* (van Poppel et al., 2008), *H. arabidopsidis ATR13* and *ATR1* (Allen et al., 2004; Rehmany et al., 2005). However, there are more virulence effectors that suppress the immunity of their hosts. For example, most of the surveyed 169 RxLR effectors of *P. sojae* could suppress cell death triggered by multiple elicitors in soybean and tobacco (Wang et al., 2011). Forty-three of 64 tested RxLR effector candidates in *H. arabidopsidis* isolate Emoy2 were able to affect plant immunity by suppressing callose deposition and facilitating bacterial growth (Fabro et al., 2011). Whereafter, a study revealed that all but one of the thirteen *RxLR* genes from isolate Waco9 of *H. arabidopsidis* could impair plant immunity (Vinatzer et al., 2014). These studies conclude that suppression of immunity is a major function of the RxLR secretome.

The oomycete *Plasmopara viticola* ([Berk. et Curt.] Berl. et de Toni) is an obligate biotroph that causes devastating downy mildew disease of grapevine. *P. viticola* is considered a typical obligate biotroph that derives all of its nutrition from living cells of grapevines via globose haustoria to complete its life cycle (Gessler et al., 2011). During the infection process, *P. viticola* can secrete a set of putative effector proteins to subvert the defense mechanism of grapevine (Casagrande et al., 2011). Preliminary search for *P. viticola* effectors in an *in vitro* germinated spore library containing 1543 cDNA clones resulted in the identification of 2 putative RxLR effectors expressed upon infection (Mestre et al., 2012). But a follow-up study about these two effectors has never been reported. A transcriptome of a *P. viticola* isolate “ZJ-1-1” was recently sequenced in our lab. Bioinformatic surveys revealed that a set of 20 RxLR-containing proteins were predicted during the infection of a *V. amurensis* “Shuanghong” grapevine (Yin et al., 2015). Then additional 11 RxLR effectors were digged out by delving further into the RNA-seq data (unpublished). Multiple alignments of the amino acid sequences of these 31 effectors showed that only PvRxLR5 and PvRxLR16 share 57.03% similarity. BLASTP searches revealed that three PvRxLR effectors (PvRxLR10, 21 and 25) from “ZJ-1-1” isolate show homology to effectors from *Bremia lactucae, H. arabidopsidis* or *P. infestans*. It seems that the majority of PvRxLR effectors are specific for *P. viticola*.

As a first step toward elucidating the molecular basis for colonization of *P. viticola* in grapevines, the *P. viticola* repertoire of candidate RxLR effectors were cloned and functionally analyzed. Out of 31 predicted RxLR effector candidates, 23 were cloned successfully. Expression patterns, subcellular localizations and their abilities to suppress cell death triggered by various elicitors were explored. Furthermore, *in planta* functional analysis revealed that *PvRxLR28* enhances plant susceptibility. Collectively, the candidate effectors identified here provide valuable information for their roles in *P. viticola* virulence.

## Materials and methods

### Plant material, strains, and growth condition

The grapevine (*V. vinifera* cv. Thompson Seedless) and tobacco (*N. benthamiana*) used in this study were grown in the greenhouse at 25°C with a photoperiod of 18 h light/6 h darkness. *Escherichia coli* and *Agrobacterium tumefaciens* strains carrying the disarmed Ti plasmid were routinely grown on Luria-Bertani (LB) agar or broth at 37°C and 28°C, respectively. *Plasmopara viticola* isolate “ZJ-1-1” was subcultured on grapevine leaf discs every 10 days at 22°C in 16/8 h light/dark cycles.

### Construction of expression plasmids

The oligonucleotides used for plasmid construction and the constructs used in this study are documented in the Supporting Information, Table S1. The *RxLR* genes were amplified using cDNA from *P. viticola* isolate “ZJ-1-1” based on the results of RNA-seq analysis (Yin et al., 2015). For the PVX assay, the open reading frames of *RxLR* genes without the predicted signal peptide were amplified using primers (Table S1). The amplified fragments were cut using appropriate restriction enzymes and ligated into the PVX vector pGR107 to generate pGR107-PvRxLR. To make the subcellular location construct pH7FWG2, 0-PvRxLR, we used the pGR107-PvRxLR as template to amplify the genes with the primers. The PCR fragments were inserted into entry vector pDONR222 and were subsequently transferred to the plant expression vector pH7FWG2, 0 with Gateway Technology (Invitrogen). To generate the stable expression recombinant vector, *PvRxLR28* PCR products were digested and ligated between *XhoI* and *BstBI* sites of a pER8 plasmid with an estrogen-inducible promoter (Zuo et al., 2000). All plasmids were validated by sequencing (Majorbio, Inc., Shanghai, China).

### RNA isolation, cDNA synthesis and quantitative RT-PCR

Grapevine leaf discs infected with spore drops of *P. viticola* “ZJ-1-1” were harvested at indicated time points and RNA was extracted using CTAB method as previously described (Iandolino et al., 2004). All cDNA Synthesis and quantitative RT-PCR reactions were conducted by using protocols established in our lab (Wu et al., 2010). The relative expression values were determined using the actin from *P. viticola* as reference gene (Schmidlin et al., 2008). Primers were designed using Beacon Designer 8.10 software with the default settings.

### *agrobacterium*-mediated transient expression in planta

Constructs were transformed into *A. tumefaciens* strain GV3101 by electroporation (Hellens et al., 2000). The pGR107-PvRxLR transformants were selected using tetracycline (12.5 μg/ml), kanamycin (50 μg/ml) and rifampicin (50 μg/ml), and the pH7FWG2,0 transformants were selected using spectinomycin (50 μg/ml) and rifampicin (50 μg/ml). To analyze the suppression of cell death triggered by different elicitors in *N. benthamiana, Agrobacteria* containing the corresponding constructs were cultured in LB medium containing 50 μg/ mL of kanamycin at 28°C with shaking at 200 rpm for 48 h. The culture was harvested and washed three times in 10 mM MgCl_2_, then resuspended in 10 mM MgCl_2_ to achieve a final OD_600_ of 0.4. For infiltration of pH7FWG2,0 into leaves, recombinant strains of *A. tumefaciens* were grown in LB medium with 50 μg/mL spectinomycin for 48 h, harvested, suspended in infiltration medium [10 mM MgCl_2_, 5 mM MES (pH 5.7), and 150 μM acetosyringone] to an OD_600_ = 0.4 before infiltration. Then 100 μL of *A. tumefaciens* cell suspension was infiltrated into leaves using a syringe without a needle. Green fluorescence and symptom development were monitored 1–2 d and 4–8 d after infiltration, respectively. *Agrobacterium*-mediated vacuum infiltration was used for grapevine transient expression assay as described (Guan et al., 2011).

### Generation of the *PvRxLR28*-transgenic *N. benthamiana*

Transgenic tobacco plants were generated by leaf-disc transformation approach (Gallois and Marinho, 1995). Briefly, leaf discs were placed into *Agrobacterium* suspension carrying a plasmid of interest for 30 min and co-cultured with bacteria for 2 d. Then the infected leaf tissue was placed adaxial side down onto a fresh shoot induction medium containing 1mg/mL of 6-BA, 30 mg/mL of hygromycin and 300 mg/mL timentin. About 1 month later, 1–2 cm tall shoots were excised and transferred into a selective rooting medium which contains 0.2 mg/L of IAA, 30 mg/mL of hygromycin and 300 mg/mL timentin. Roots were generated within 2–3 weeks in culture and transferred to soil for seeds collected. All plants were kept in growth cabinet at 25°C with a 16 h light and 8 h dark regime. T2 transforments were treated with 17-β- estradiol (100μM) and Silwet L-77 (0.01%) 48 h prior to conducting further experiments.

### Pathogen infection assays

For *P. viticola* infection, 2 days post-infiltration grapevine leaf discs were inoculated with 30 μL of spore suspensions with a concentration of 10^5^ spores/ mL on the abaxial surface. Infected disc samples were kept in a growth cabinet at 22°C with a 16 h photoperiod. Phenotype was monitored within 4–5 d.

For *Phytophthora parasitica* infection, two approaches were used as described (Rajput et al., 2014). Briefly, *P. parasitica* was grown on 10% (v/v) V8 juice agar at 25°C in the dark and zoospores were prepared as reported previously (Zhang et al., 2012). For detached leaves, 20 μL of zoospore suspensions with a concentration of 100 zoospores/μL were applied onto the abaxial side of detached *N. benthamiana* leaves. The phenotype was monitored within 72 h, and photographs were taken at 36 h post-inoculation. For whole seedlings, the infection assays were performed by using the root-dip inoculation method. The whole transgenic plants were inoculated with zoospores. The GFP-transgenic lines were used as controls. The inoculated plants were maintained in a moist chamber, and disease progression was monitored within 10 days.

### Protein extraction and western blot

*N. benthamiana* leaf tissues were ground in liquid nitrogen and mixed with an equal volume of cold protein extraction buffer [50 mM HEPS, 150 mM KCl, 1 mM EDTA (pH 8.0), 0.1% triton X-100, 1 mM DTT, 1 × Protease Inhibitor Cocktail (Sigma)]. Suspensions were mixed and centrifuged at 12,000 rpm for 15 min at 4°C. The supernatant was transferred to a new tube and boiled in 5 × SDS loading buffer. Proteins were separated by 12% SDS-PAGE and transferred to a nitrocellulose blotting membrane. The membrane was washed with PBST for 15 min and then blocked in 5% non-fat milk for 1 h. Mouse monoclonal anti-Flag antibody (Transgen Biotech) was added at a ratio of 1:2000 and incubated overnight at 4°C, followed by three washed with PBST. Then the membranes incubated with goat anti-mouse lgG (H&L)-HRP polyclonal antibody (Jiamay Biotech) for 1 h at a ratio of 1: 5000 at RT. After three washes with PBST, the membranes were visualized using HRP-ECL system.

### Confocal microscopy

To analyze the subcellular localizations of the PvRxLRs in *N. benthamiana*, patches cut from leaf samples were immersed into PBS buffer which contains 5 mg/L 4′, 6-diamidino-2-phenylindole (DAPI) for staining the nuclei for 10 min. Then the patches were mounted on microscope slides and observed with a Nikon C1 Si/TE2000E confocal laser scanning microscope. For co-localization, co-infiltrated leaves with *Agrobacterium* contain pH7FWG2,0-PvRxLR55-GFP and pBI121- Avh241-mCherry constructs were detected at 2 dpi. The excitation/emission wavelengths for GFP, DAPI and RFP were 488 nm/505–530 nm, 405 nm/420–480 nm and 543 nm/600–630 nm, respectively. EZ-C1 3.00 software was used for image processing.

### Analysis of H_2_O_2_ level

H_2_O_2_ was monitored *in situ* using 3, 3-diaminobenzi-dine (DAB) (Sigma) staining as described previously (Thordal-Christensen et al., 1997). Briefly, the infected leaves 12 h after inoculation were soaked in DAB solution at 1 mg/mL and maintained for 8 h at 25°C. Then the samples were transferred into 95% ethanol and boiled for 15 min until the chlorophyll was completely bleached. The bleached leaves were further soaked in 2.5 g/mL trichloracetic aldehyde solution to clear the background. The H_2_O_2_ level was quantified by Image J and Photoshop.

## Results

### Expression patterns of candidate RxLR effector genes of *p. viticola*

**Figure 1:**
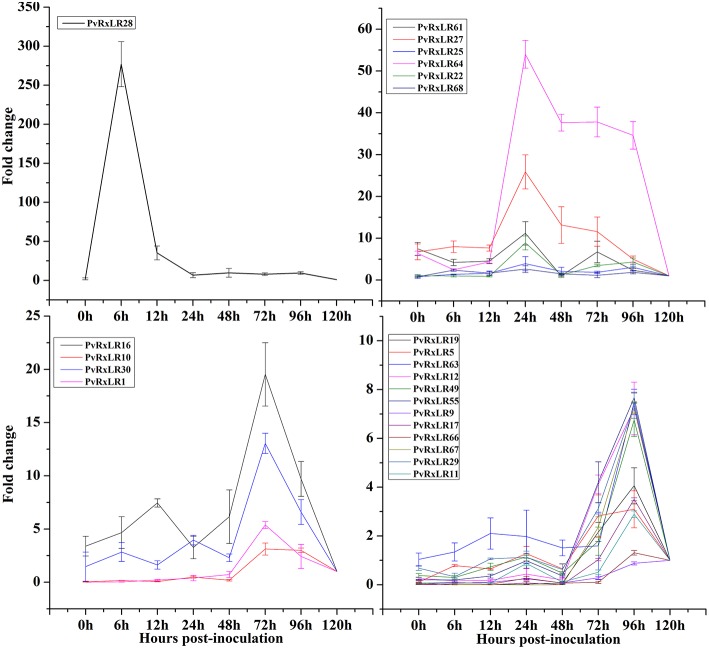
**Expression patterns of 23 ***PvRxLR*** effector genes from ***P. viticola*** isolate “ZJ-1-1” during the pathogen-grapevine infection**. Relative *PvRxLRs* mRNA levels were quantified by quantitative RT-PCR in samples and grapevine leaves inoculated with *P. viticola* at different time-points post infiltration. *P. viticola* actin transcripts were used as a reference. Error bars represent standard errors (SEs) from three biological replicates.

When the compatible *P. viticola* strain “ZJ-1-1” was inoculated onto the“downy mildew-resistant” *V. amurensis* “Shuanghong” grapevine, the disease progressed quickly on the infected leaves, and branched hyphae with many haustoria and sporangia were visible by 3 days post-inoculation (Li et al., 2015a). When the transcriptional levels of effector genes were measured at different time courses during the infection (0, 6, 12, 24, 48, 72, 96, and 120 hpi), all the 23 *PvRxLR* genes identified from the *P. viticola* strain “ZJ-1-1” were up-regulated at some time points. However, the expression patterns, which could be grouped into four different kinds, varied greatly among these effectors (Figure 1). In general, the *PvRxLR* genes of the first two groups were highly expressed at the earlier infection stages (6 and 24 hpi), while in the other two groups, increasing gene expressions occurred much latter (72 and 96 hpi). *PvRxLR28*, for example, increased more than 275-fold at 6 hpi, and then declined sharply at the subsequent interacting time points. A similar pattern of induction was described for RxLR effectors of *H. arabidopsidis* and *P. sojae* which has been termed “immediate-early, low” (Wang et al., 2011; Anderson et al., 2012).

### Subcellular localizations of PvRxLRs in *n. benthamiana*

**Figure 2:**
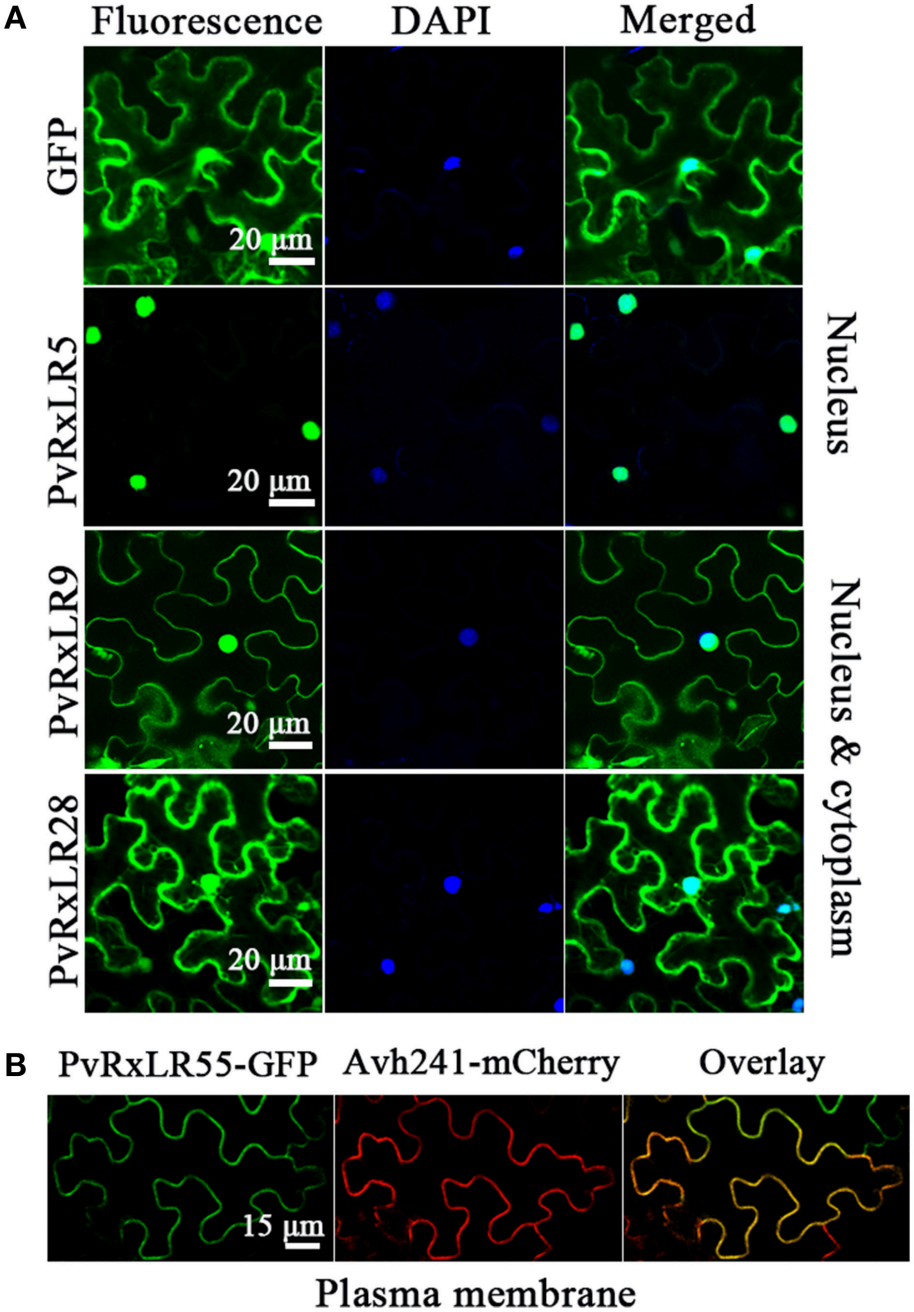
**Subcellular localizations of PvRxLR proteins. (A)** *N. benthamiana* leaf tissues showing expression of the PvRxLRs-GFP fusion protein driven by the 35S promoter. Scale bar = 20 μm. **(B)** Co-localization of PvRxLR55 protein with plasma membrane marker Avh241. PvRxLR55-GFP showing GFP signal merges completely with Avh241-mCherry. GFP fusion to the PvRxLR55 protein is shown in green, Avh241-mCherry in red and overlay of the two proteins in dark field view. Scale bar = 15 μm.

The localizations of these pathogen effectors after entering a plant cell could well be an indication of their mode of action (Dowen et al., 2009; Schornack et al., 2010). To determine the subcellular localizations of PvRxLRs, we generated green fluorescent protein-tagged versions of the 23 effectors downstream of their signal peptide cleavage site. Fusion plasmids were expressed transiently in *N. benthamiana* using agroinfiltration. Results showed that 6 effector proteins strictly localized to the nucleus, while 16 of them localized to both nucleus and the cytoplasm (Figure 2A, Figure S1). The only exception is the effector PvRxLR55 that was only detected in the cell membrane. For further confirmation, co-localization of PvRxLR55-GFP has been done with a plasma membrane protein Avh241 (Yu et al., 2012). It was observed that the GFP signal of PvRxLR55-GFP overlapped with Avh241—mCherry fluorescence signal, suggesting the localization of PvRxLR55 to the plasma membrane (Figure 2B).

### PvRxLRS suppress different elicitors-induced cell death in *n. benthamiana*

**Figure 3:**
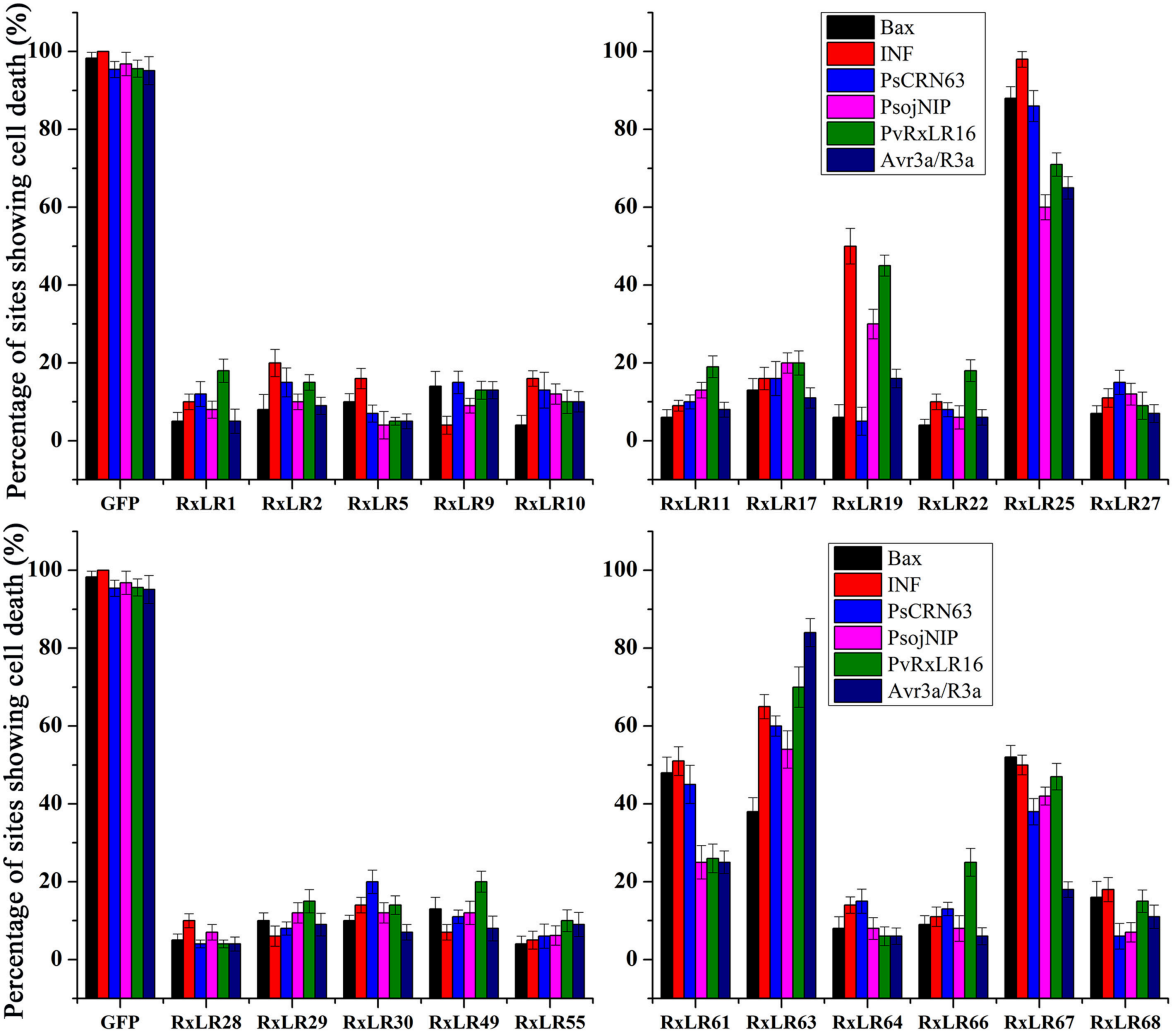
**Suppression of different elicitors-induced cell death in ***N. benthamiana*** by PvRxLR effectors**. *Agroinfiltration* sites in each *N. benthamiana* leaf expressing *PvRxLRs* and *GFP* (negative control) were challenged after 12 h with *A. tumefaciens* carrying the indicated elicitors. Experiments were repeated three times with 8 sites for each elicitor or GFP and assessed with percentage of cell death sites 5 d after cell death inducer infiltration.

Virulence of the effectors was assessed by testing their abilities of inhibiting programmed cell death in tobacco using transient over-expression. The elicitors used in the present study included the mouse BAX (Lacomme and Santa Cruz, 1999), the PAMP elicitor INF1 from *P. infestans* (Kamoun et al., 1998), the *P. sojae* effector CRN63 (Liu et al., 2011), the necrosis-inducing protein PsojNIP (Qutob et al., 2002) and the Avr3a/R3a (Armstrong et al., 2005). The PvRxLR16 of *P. viticola* was also used as an elicitor as it could induce cell death in *N. benthamiana*. *Agrobacterium* strains carrying each effector gene were infiltrated into *N. benthamiana* leaves 12 h prior to infiltration of the cell death-inducers. Of the 22 effectors tested, 17 effector proteins completely inhibited cell death induced by the above elicitors, while the remaining five (PvRxLR19, PvRxLR25, PvRxLR61, PvRxLR63, and PvRxLR67) partially suppressed the tobacco PCD (Figure 3, Figure S2). As expected, the negative control of GFP alone did not suppress the PCD. Notably, our results are consistent with previous findings that seven effectors (PvRxLR1, 5, 9, 10, 11, 19, 25) could repress BAX- and INF-induced cell death (Yin et al., 2015). These results suggest that each of the 17 effectors may act as a broad suppressor of cell death to interrupt plant immunity.

### The C-terminus and the nuclear localization are required for mediating the suppression of PCD

**Figure 4:**
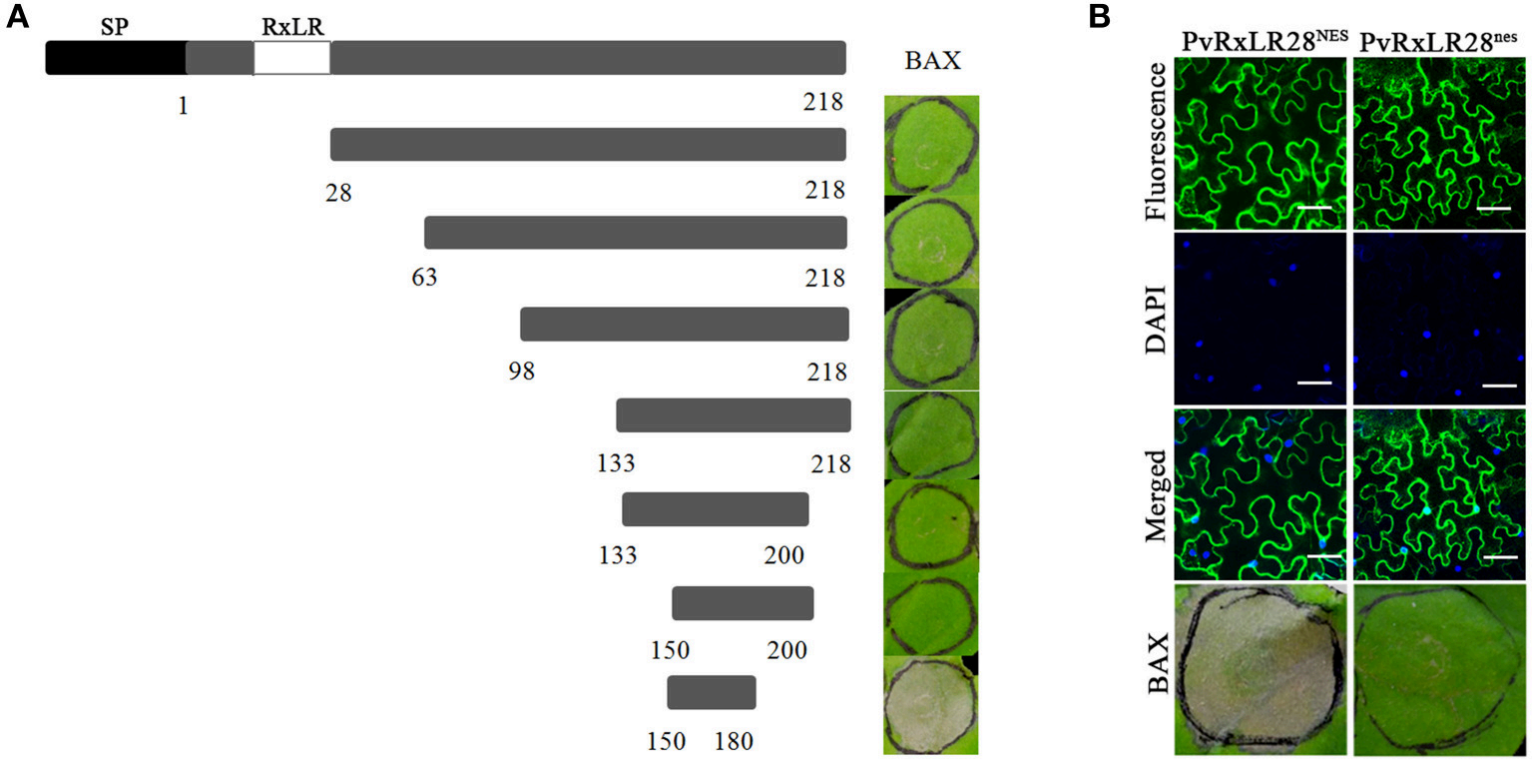
**The C-terminus and the nuclear localization are essential for the function of PvRxLR28 effector. (A)** Deletion analysis of PvRxLR28. Deletion mutants were expressed by agroinfiltration in *N. benthamiana* to assay suppression of cell death induced by BAX. Typical symptoms were photographed 5 d after infiltration of the BAX. **(B)** Nuclear localization is required for mediating the suppression of PCD. NES impairs accumulation of GFP: PvRxLR28 in *N. benthamiana* nucleus. *N. benthamiana* leaves were agroinfiltrated with the indicated constructs 48 h before assessment of GFP confocal imaging. Scale bars, 50 μm. NES and nes represent the nuclear export signal and nonfunctional NES respectively. Scale bar = 30 μm.

To understand how the PvRxLRs work, effector PvRxLR28, the most dramatic up regulating one triggered by pathogen- host interaction was chosen for study in detail. Seven deletion mutants of PvRxLR28 were generated and tested for their abilities to inhibit BAX-induced PCD. Figure 4A demonstrated that positions from 150 to 200 of PvRxLR28 were required for suppressing the cell death. The result also indicated that the conserved N-terminal RxLR motif was not necessary for the PCD suppressions.

In order to determine whether the localization is crucial for PvRxLR28 to suppress PCD, a synthetic NES (nuclear exclusion signal) and a nes (nonfunctional NES) were added to the C terminus, respectively. Cell death symptoms were observed when the NES was attached to PvRxLR28, but not with the attachment of the nes (Figure 4B). It is clear that the ability of PvRxLR28 to repress cell death relies on the nuclear localization.

### *PvRxLR28* expression enhances plant susceptibility to pathogens

**Figure 5:**
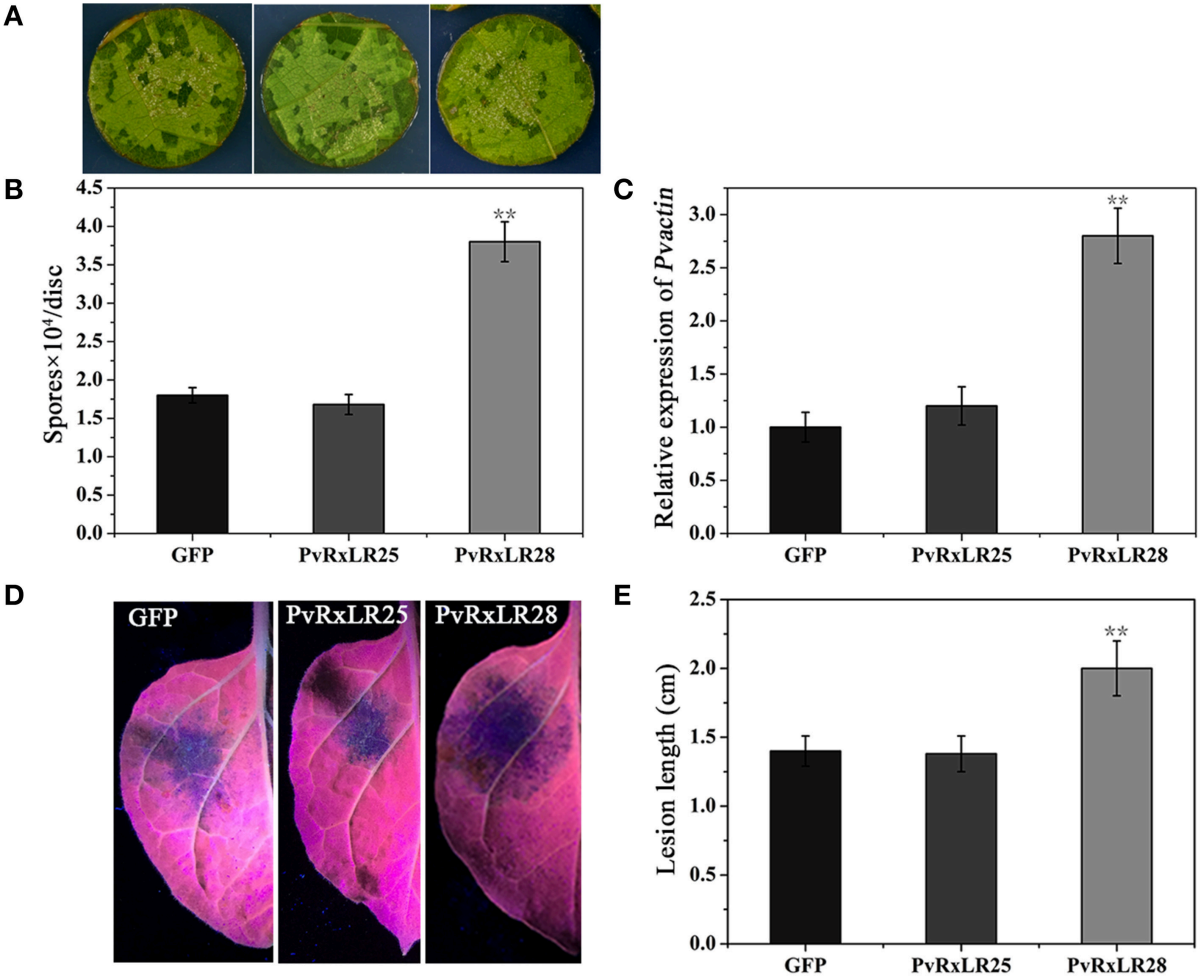
**Suppression of the plant resistance by transient expressing ***PvRxLR28***. (A)** Phenotypes of grapevine leaf discs expressing *GFP, PvRxLR25* or *PvRxLR28*. The representative pictures were taken at 5 d post infection of *P. viticola*. **(B)** Average zoospore numbers of *P. viticola* on a total of 50 infected discs. Error bars represent SD from three independent biological replicates (^**^P < 0.01, Dunnett's test). **(C)** The transcript accumulation of *P. viticola* actin. The development of pathogen was calculated by qRT-PCR assays of *P. viticola* actin at 5 dpi. Transcript level of grapevine actin gene was used as an internal reference (^**^P < 0.01, Dunnett's test). **(D)** Lesions of the *N. benthamiana* leaves expressing *GFP, PvRxLR25* or *PvRxLR28* at 36 hpi. **(E)** Lesion diameters of *N. benthamiana* leaves expressing the indicated genes inoculated with *P. parasitica.* Statistical analyses were performed using a Dunnett's test (^**^P < 0.01).

As PCD is a hallmark of HR-based immunity in plants (Abramovitch et al., 2003), we dissected the effects of *PvRxLR28* expression on plant immunity. GFP and effector PvRxLR25, which could not suppress cell death induced by elicitors, were chosen as controls. *PvRxLR28* was transiently expressed in the leaves of grapevine and tobacco 2 days prior inoculation of *P. viticola* and *P. parasitica* zoospores, respectively. *P. viticola* development was evaluated by monitoring zoospore numbers and *PvActin* transcript accumulation in infected leaf discs. *P. parasitica* development was evaluated by lesion length on leaf areas. More zoospores of *P. viticola* were produced in *PvRxLR28* - expressing grapevine leaves, and higher expression of actin transcripts was also observed compared to controls (Figures 5A–C). Similarly, the lesion diameter was significantly larger in *PvRxLR28-* than *PvRxLR25-* or *GFP*-expressing tobacco (Figures 5D,E). These results show that transient expression of *PvRxLR28* could impair plant resistance to pathogens.

**Figure 6:**
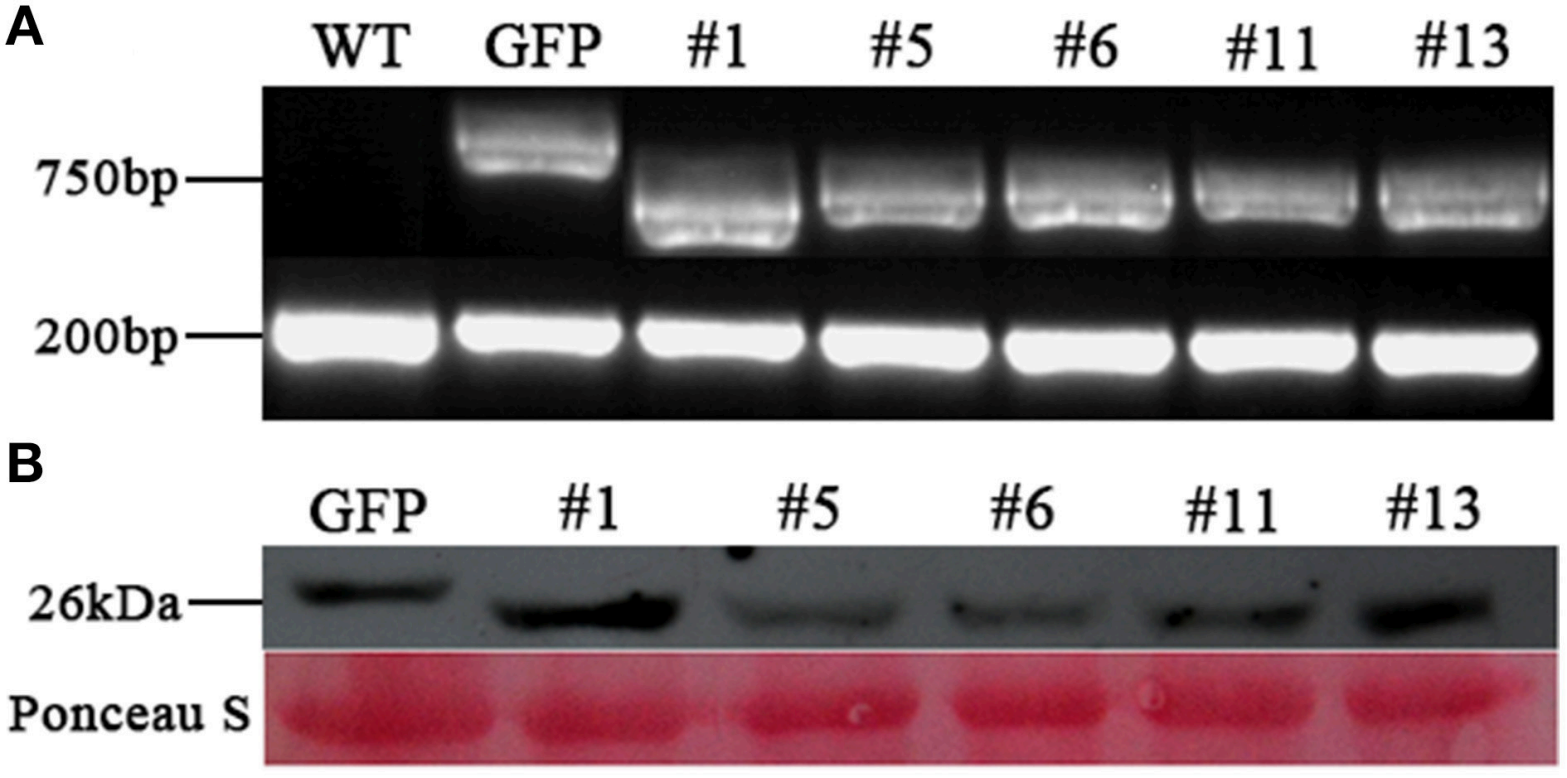
**Expression analysis of ***PvRxLR28*** in transgenic ***N. benthamiana.*** (A)** RT-PCR analysis of *PvRxLR28* expression in transgenic lines of T2 generation, and *EF1-*α was used as a control. **(B)** Western blot analysis of total proteins from transgenic *N. benthamiana* lines using monoclonal antibody against flag. The leaves for RT-PCR and western blot were collected at 2d post spraying of 17-β- estradiol (100 μM) and Silwet L-77 (0.01%).

**Figure 7:**
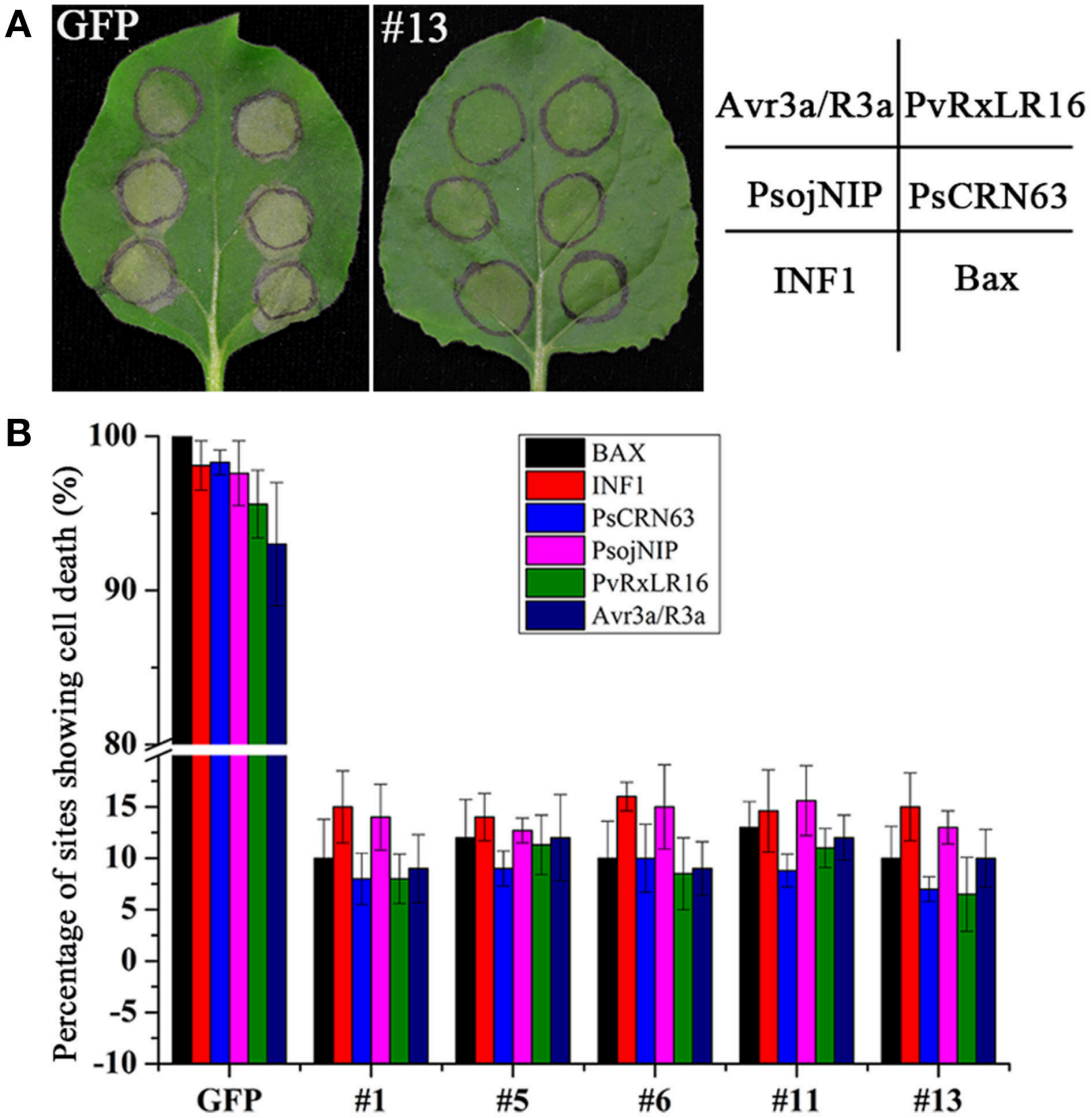
**Suppression of cell death in ***PvRxLR28***- transgenic ***N. benthamiana.***** **(A)** Phenotypes of leaves challenged with cell death inducers 5 d after infiltration. **(B)** Showing percentages of cell death sites on the *PvRxLR28*- transgenic lines assessed from 16 infiltrated leaves of three independent experiments.

**Figure 8:**
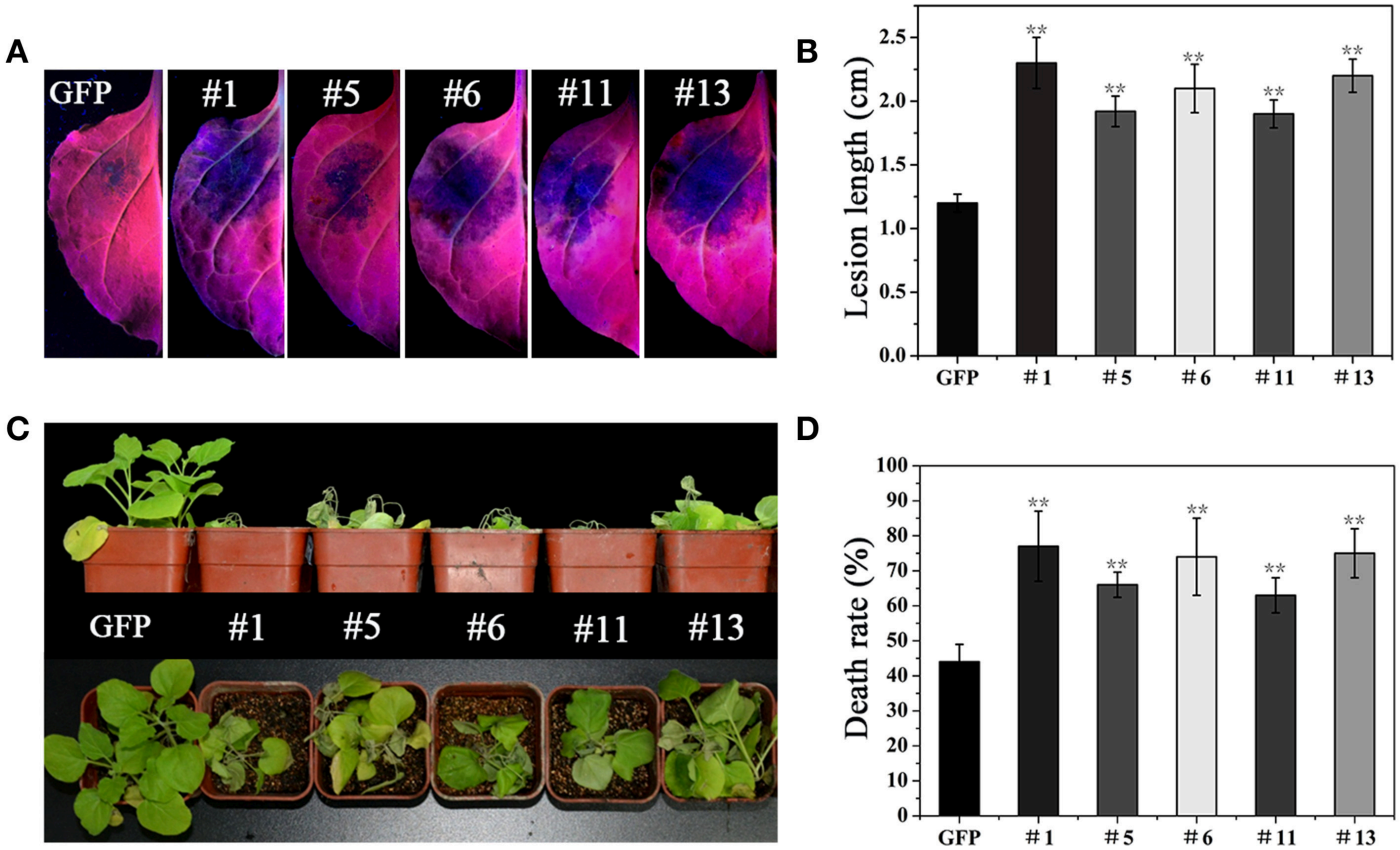
**Suppression of the resistance in ***PvRxLR28***-transgenic ***N. benthamiana.*** (A)** Detached leaves of *GFP*- and *PvRxLR28*-transgenic plants 36 h post inoculated with *P. parasitica* zoospores. **(B)** Average lesion diameters of the inoculated leaves at 36 hpi. Error bars represent standard deviation calculated from 12 independent biological replicates (^**^P < 0.01, Dunnett's test). **(C)** The transgenic lines inoculated with zoospores of *P. parasitica* using the root dip method at 4 dpi. **(D)** Death rates of transgenic plants inoculated with *P. parasitica*. The experiments were repeated four times with similar results. 20 plants for each line were used for each treatment in each experiment. Bars represent the standard errors. Asterisks indicate statistical significance (^**^P < 0.01, Dunnett's test).

To verify the above observation, we generated a *PvRxLR28: Flag* fusion construct driven by an estrogen-inducible promoter, and introduced it into *N. benthamiana* leaf discs by *Agrobacterium*-mediated transformation. A total of 35 independent transgenic lines (T0) including 21 *PvRxLR28*-expressing plants and 14 GFP- expressing plants were obtained. T1 lines were generated by self-pollination. RT-PCR and Western-blot analysis confirmed that *PvRxLR28* and *GFP* were highly expressed in several T2 lines after induced by oestradiol (Figure 6). Similar to transient expression assay, stable *PvRxLR28*-transgenic plants also blocked cell death triggered by the six elicitors (Figure 7). When the transgenic plants were challenged with oomycete *P. parasitica*, detached leaf tissues of *PvRxLR28*-transgenic plants developed larger lesion than the ones of *GFP* lines (Figures 8A,B). When they were sprayed with *P. parasitica*, the *PvRxLR28*-transgenic plants also showed earlier and severer symptoms with higher death rate than control lines even though both *PvRxLR28*- and *GFP*-transgenic plants showed symptoms of wilting and stunting (Figures 8C,D). The above findings clearly indicated that effector PvRxLR28 enhanced plant susceptibility to oomycete pathogens.

### *PvRxLR28* expression reduces the transcriptional levels of the defense-related genes and impairs the H_2_O_2_ accumulation in *n. benthamiana*

**Figure 9:**
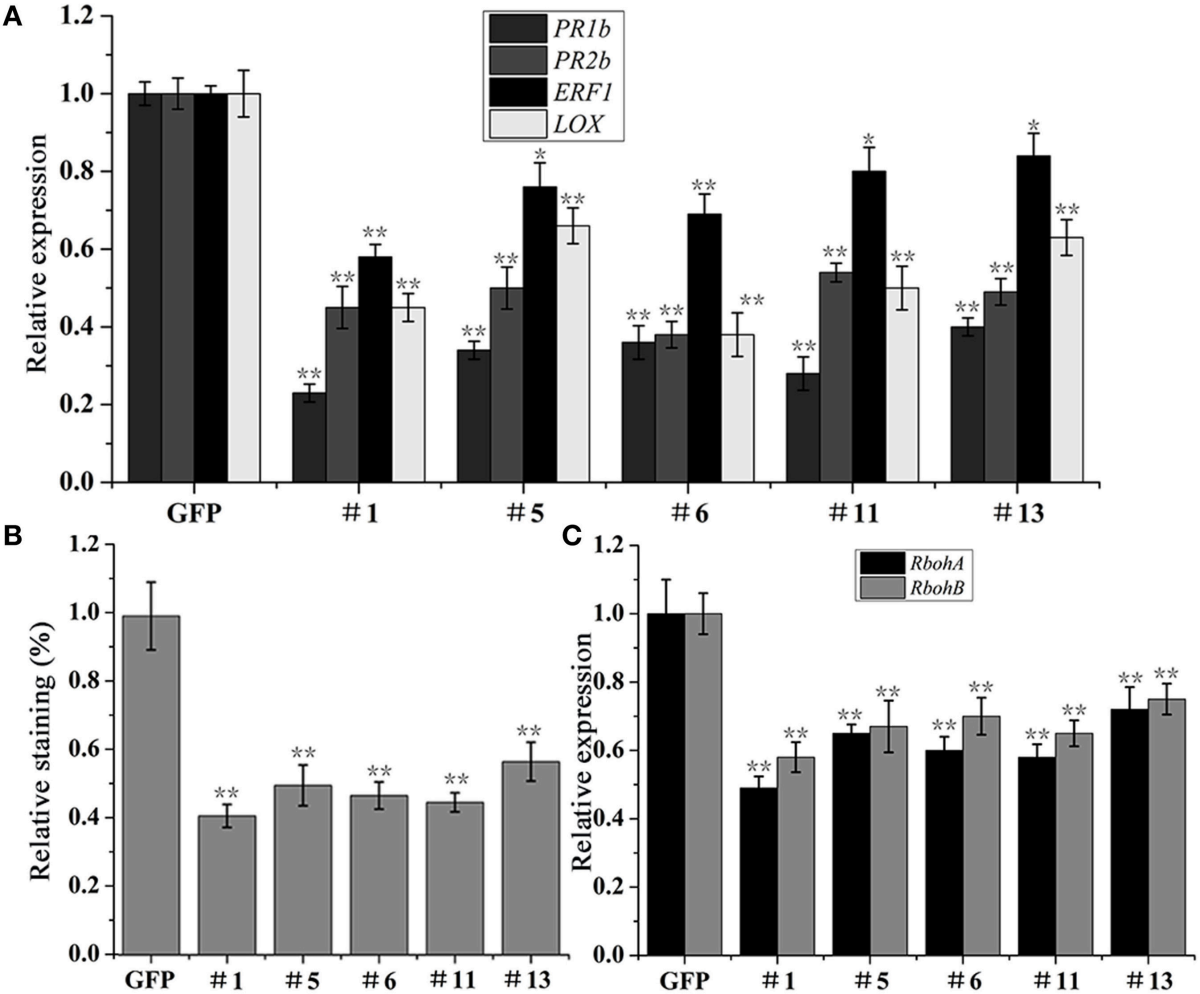
**Suppression of pathogenesis-related genes and H_**2**_O_**2**_ accumulation in ***N. benthamiana*** by PvRxLR28. (A)** Relative expression levels of *PR1b, PR2b, ERF1, LOX* genes in the transgenic lines at 36 dpi with *P. parasitica* zoospores. The relative expression levels were normalized to the *EF1*α gene. Error bars represent standard errors (^**^P < 0.01, ^*^P < 0.05, Dunnett's test). **(B)** Relative intensity of DAB staining in *P*. *parasitica*-infected *N. benthamiana*. Asterisks indicate significant differences (^**^P < 0.01, Dunnett's test). **(C)** Relative expression levels of ROS-producing genes *RbohA* and *RbohB*. Asterisks indicate significant differences (^**^P < 0.01, Dunnett's test).

To preliminarily explore the role of PvRxLR28 in plant immune response, expressional levels of selected host defense-related genes were examined using qRT-PCR. Since salicylic acid (SA), jasmonic acid (JA) and ethylene (ET) are three main phytohormones essential for the immune response against pathogen (Vlot et al., 2009; Ballaré, 2011; Robert-Seilaniantz et al., 2011). Marker genes *PR1b* and *PR2b* (SA), *LOX* (JA) and *EFR1* (ET), in each of these phytohormone mediated signaling pathways, were analyzed. These genes all positively contribute to the resistance against pathogen. Expression levels of these four defense-related genes were significantly decreased in *PvRxLR28*-transgenic lines during *P. parasitica* infection (Figure 9A), suggesting that PvRxLR28 may enhance plant susceptibility by repressing the expression of the defense-related genes in plants.

To expand understanding of the mechanism that leads to host susceptibility to pathogen, we also analyzed the accumulation of H_2_O_2_ in *N. benthamiana* leaves which takes part in the defense responses (Thordal-Christensen et al., 1997). The H_2_O_2_ levels in the transgenic plants were measured at the early infective stages of *P. parasitica* using DAB staining method. As a result, the relative staining was significantly lower in the *PvRxLR28*-transgenic lines than that in the *GFP* lines (Figure 9B). In order to elucidate the possible mechanisms underlying the reduced H_2_O_2_ accumulation in *PvRxLR28*-transgenic plants, the expression levels of *RbohA* and *RbohB* genes that encode ROS-producing proteins were also measured (Figure 9C). Expression of these genes was significantly reduced in the stable *PvRxLR28*-transgenic plants and thus the H_2_O_2_ reduction might be caused by down-regulation of ROS-producing genes. In conclusion, PvRxLR28 effector enhancing plant susceptibility is likely by reducing the transcriptional levels of the defense-related genes and impairing the H_2_O_2_ accumulation.

## Discussion

Despite the worldwide economic impact of diseases caused by *P. viticola*, little is known about the molecular basis of the pathogenicity of this species. The study of the secrete proteins from the pathogen may greatly advance our understanding of pathogen virulence. In the secretome of *P. viticola* isolate “ZJ-1-1,” a total of 31 putative RxLR effectors have been predicted. Here we used a virus *in planta* over-expression system to assess the potential of PvRxLRs to manipulate immune response in non-host plant. The utility of the screening approach has been validated in studies of effectors from *Phytophthora sojae* (Wang et al., 2011), *Globodera rostochiensis* (Ali et al., 2015) and *Valsa mali* (Li et al., 2015b). Of 23 *PvRxLR* effector genes cloned (Table S2), only *PvRxLR16* could directly trigger cell death, while 17 were able to completely and 5 partially suppress PCD induced by various elicitors. Findings from this study strongly suggest that *P. viticola* secreted RxLR effectors to suppress PTI, ETI and other types of host immune responses during the pathogen infection. Although the mechanism of broad PCD/defense suppression by *P. viticola* effectors is not clear, it is possible that they target a central component downstream from the convergence node of resistance network, or multiple distinct pathways.

To confirm the roles of effectors in plant immunity, PvRxLR28 was chosen for further research. As *P. viticola* was recalcitrant to genetically manipulate because of its obligate lifestyle, the effector PvRxLR28 was expressed in plant cells by transiently and stably integrated transgenes. Overexpression of the *PvRxLR28* in tobacco and grapevine enhanced their susceptibility to *P. parasitica* and *P. viticola*, respectively, suggesting that this effector contributes to pathogen virulence. The H_2_O_2_accumulation and the defense marker genes in the transgenic plants were significantly lower than in the control lines, indicating PvRxLR28 can promote pathogen infection by reducing H_2_O_2_levels and suppressing SA and JA signaling pathways which usually act antagonistically in plant defense. Due to the difficulty of obtaining stable transgenic grapevines, the effector candidate PvRxLR28 was stably expressed in non-host *N. benthamiana* in the current study. Therefore, a more convincing conclusion about the enhancement of host susceptibility to the pathogen by PvRxLR effectors should come from a transgenic grapevine experiment.

It has been documented that N-terminal RxLR motif is involved in translocation mechanism, while the C-terminus is important for function (Whisson et al., 2007; Dou et al., 2008). A series of deletion mutants of PvRxLR28 indicated that the C-terminus of PvRxLR28 is essential for suppressing PCD, although our present data could not demonstrate that the RxLR is responsible for entering the plant cell. This unique characteristic of RxLR effectors seems conserved in different oomycete pathogens. Similar to previous reports (Boch and Bonas, 2010; Kelley et al., 2010; Caillaud et al., 2013; Zheng et al., 2014), all the tested PvRxLR effectors except PvRxLR55 could localized to the plant cell nucleus, implicating that most RxLR effectors of *P. viticola* tested in this study may function by manipulating the host nuclear processes to attenuate the plant defense and subsequently promote pathogen infection.

Interestingly, the percentage of putative effectors that can suppress elicitor-induced PCD varied a good deal among different species. For example, in a study of *V. mali* effectors, only 7 out of 70 randomly selected effectors exhibited the capacity to suppress BAX-induced PCD (Li et al., 2015b), while 107 of 169 RxLR effectors from *P. sojae* (Wang et al., 2011), and 43 of 64 RxLR effectors from *H. arabidopsidis* (Fabro et al., 2011) could suppress cell death induced by BAX or plant immunity. It seems likely that the pathogens whose lifestyle contains biotrophic phase may have a high percentage of effectors which can suppress PCD or immune response. Our study showed that 22 of 23 *P. viticola* effectors can completely or partially suppress PCD in *N.benthamiana*, which are consistent with the hypothesis. It also appears that there is a high degree of functional redundancy in effectors suppressing PCD in these pathogens. The molecular basis of this functional redundancy bears further investigation.

In conclusion, results from this study clearly indicate that majority of the tested PvRxLR effectors could suppress PCD in *N. benthamiana*, and thus they may make primary contribution to pathogen virulence. In addition, functional studies of PvRxLR secretome will ultimately help to elucidate the mechanisms underlying the pathogenicity of *P. viticola* to grapevine.

## Author contributions

YZ and JL conceived the research. JL, JX, and XL designed the research. JX, XL, LY, and JW performed the experiments. JL, JX, and XL wrote the article.

### Conflict of interest statement

The authors declare that the research was conducted in the absence of any commercial or financial relationships that could be construed as a potential conflict of interest.

## Supplementary material

Table S1 **Primers used in this study**.

Table S2 **Genbank accession numbers and amino acid sequences of the PvRXLRs**.

Figure S1 **Subcellular localizations of PvRxLRs in ***N. benthamiana*** leaf tissues**.

Figure S2 **Suppression of different elicitors-triggered cell death in ***N. benthamiana*** by PvRxLR effectors**.
