# Supplementary material for: Studying the Mechanism of Plasmopara viticola RxLR Effectors on Suppressing Plant Immunity
Source: Front Microbiol. 2016 May 18;7:709. doi: 10.3389/fmicb.2016.00709 (PMC4870276; doi:10.3389/fmicb.2016.00709)
Supplement: Table S1 — Primers used in this study. [file Table1.DOCX]

*Supplementary Material*

**Screening RxLR effectors** **of *Plasmopara viticola* for suppressing plant immunity**

Jiang Xiang^1§^, Xinlong Li^1^^§^, Jiao Wu^1^, Ling Yin^2^, Yali Zhang^1^, Jiang Lu^1*^

***** Corresponding Author: Jiangluvitis@cau.edu.cn

^1^ The Viticulture and Enology Program, College of Food Science and Nutritional Engineering, China Agricultural University, Beijing, China.

^2^ Guangxi Crop Genetic Improvement and Biotechnology Laboratory, Guangxi Academy of Agricultural Sciences, Nanning, China.

**Supplementary Table**

Table S1: Primers used in this study

| Primer name | Primer sequence | Purpose or vector |
| --- | --- | --- |
| *PvRXLR1-ClaI-F*  *PvRXLR1-SalI-R* | CCatcgatATGCTGGGAGAC | pGR107 |
|  | ACGCgtcgacTTAATGACGCGT |  |
| *PvRXLR2-ClaI-F*  *PvRXLR2-SalI-R* | CCatcgatATGCTGGAGATT | pGR107 |
|  | ACGCgtcgacTTAAGGCCCCCT |  |
| *PvRXLR5-ClaI-F*  *PvRXLR5-SalI-R* | CCatcgatATGATTGACCAA | pGR107 |
|  | ACGCgtcgacCTAATGTGTAAC |  |
| *PvRXLR9-ClaI-F*  *PvRXLR9-SalI-R* | CCatcgatATGAGCAGCTCT | pGR106 |
|  | ACGCgtcgacTCACCTGCGTTG |  |
| *PvRXLR10-ClaI-F*  *PvRXLR10-SalI-R* | CCatcgatATGTCCTCGCCA | pGR107 |
|  | ACGCgtcgacTTACTCAGAGCT |  |
| *PvRXLR11-ClaI-F*  *PvRXLR11-XmaI-R* | CCatcgatATGACGAACGCC | pGR107 |
|  | TCCCcccgggTCAATGAGCGAC |  |
| *PvRXLR16-ClaI-F*  *PvRXLR16-SalI-R* | CCatcgatATGACAGGAAAT | pGR107 |
|  | ACGCgtcgacCTACCTATTGAAT |  |
| *PvRXLR17-ClaI-F*  *PvRXLR17-SalI-R* | CCatcgatATGTCGGATAAG | pGR107 |
|  | ACGCgtcgacCTAATATTCCTT |  |
| *PvRXLR19-AscI-F*  *PvRXLR19-NotI-R* | TTggcgcgccATGGTAGCCGAC | pGR107 |
|  | ATAAGAATgcggccgcTTACCCTTGAAG |  |
| *PvRXLR22-ClaI-F*  *PvRXLR22-SalI-R* | CCatcgatATGAATGAAGTT | pGR107 |
|  | ACGCgtcgacCTAATCTTGCCG |  |
| *PvRXLR25-XmaI-F*  *PvRXLR25-SalI-R* | TCCcccgggATGGTTAACCGAGCCA | pGR107 |
|  | ACGCgtcgacTTACAACTTCCCGTAAAC |  |
| *PvRXLR27-ClaI-F*  *PvRXLR27-SalI-R* | CCatcgatATGGCCGACGAAGTCAAC | pGR107 |
|  | ACGCgtcgacTTAGAAGATTATTAGGAG |  |
| *PvRXLR28-ClaI-F*  *PvRXLR28-SalI-R* | CCatcgatATGGCTTGGGGA | pGR107 |
|  | ACGCgtcgacCTACTCGAAGAAT |  |
| *PvRXLR30-ClaI-F*  *PvRXLR30-SalI-R* | CCatcgatATGGTTGCGACT | pGR107 |
|  | ACGCgtcgacTCATTTGGCCAC |  |
| *PvRXLR49-ClaI-F*  *PvRXLR49-SalI-R* | CCatcgatATGCTGGATGAT | pGR107 |
|  | ACGCgtcgacTTAAACGCTTCG |  |
| *PvRXLR54-ClaI-F*  *PvRXLR54-SalI-R* | CCatcgatATGCACGTCTTC | pGR107 |
|  | ACGCgtcgacTCAAGCAGTGCG |  |
| *PvRXLR55-ClaI-F*  *PvRXLR55-SalI-R* | CCatcgatATGTATGGCATC | pGR107 |
|  | ACGCgtcgacTTACTTGTTCAT |  |
| *PvRXLR61-XmaI-F*  *PvRXLR61-SalI-R* | TCCCcccgggATGTATCCCTCACAAATT | pGR107 |
|  | ACGCgtcgacCTAATGGCAGCTTGGTAT |  |
| *PvRXLR63-ClaI-F*  *PvRXLR63-SalI-R* | CCatcgatATGCACCATATA | pGR107 |
|  | ACGCgtcgacCTACTTCCAGCA |  |
| *PvRXLR64-ClaI-F*  *PvRXLR64-SalI-R* | CCatcgatATGCAACCAAGC | pGR107 |
|  | ACGCgtcgacTTACATCCAAAAG |  |

| Primer name | Primer sequence | Purpose or vector |
| --- | --- | --- |
| *PvRXLR66-ClaI-F* | CCatcgatATGCTGACGAGC | pGR107 |
| *PvRXLR66-SalI-R* | ACGCgtcgacTTAATTTTCGGT |  |
| *PvRXLR67-ClaI-F* | CCatcgatATGAGCAGTGGT | pGR107 |
| *PvRXLR67-SalI-R* | ACGCgtcgacTTAACAGAATGC |  |
| *PvRXLR68-ClaI-F* | CCatcgatATGGCCCCCTTC | pGR107 |
| *PvRXLR68-SalI-R* | ACGCgtcgacTCACGAAGCCGA |  |
| *PvRXLR1-qRT-F* | GAGTGTGGATAACAAGAG | qRT-PCR |
| *PvRXLR1-qRT-R* | TTATTTAAGAAGCCTCGTT |  |
| *PvRXLR2-qRT-F* | GAAGAACGATCTGCTCTG | qRT-PCR |
| *PvRXLR2-qRT-R* | CAATCCGTGGAATGAGAA |  |
| *PvRXLR5-qRT-F* | ATTTGGCAGACTTTGAAA | qRT-PCR |
| *PvRXLR5-qRT-R* | TGGAGACTATGGAAATACT |  |
| *PvRXLR9-qRT-F* | TGGAACTGACGGGAATGTA | qRT-PCR |
| *PvRXLR9-qRT-R* | TGGGCATGTGAGGGTTTA |  |
| *PvRXLR10-qRT-F* | GGGATTTAAGAAAGGAGTATT | qRT-PCR |
| *PvRXLR10-qRT-R* | CTCTTCTCGTCGTAGTAA |  |
| *PvRXLR11-qRT-F* | AAGCGACGACTCTACTCA | qRT-PCR |
| *PvRXLR11-qRT-R* | CCTGCCATTGACATACGG |  |
| *PvRXLR16-qRT-F* | ACCCGCAATTACCGAAAT | qRT-PCR |
| *PvRXLR16-qRT-R* | TACATCAGTACCGCCTCT |  |
| *PvRXLR17-qRT-F* | TCGCTAAGAACTCGTGAA | qRT-PCR |
| *PvRXLR17-qRT-R* | GACCTATCCTCCTCAACTT |  |
| *PvRXLR19-qRT-F* | GCATTGGCGTTACTATCT | qRT-PCR |
| *PvRXLR19-qRT-R* | GGCTTAGTTGGACATCTC |  |
| *PvRXLR22-qRT-F* | GATTCTGGTATCCGTATTACAACT | qRT-PCR |
| *PvRXLR22-qRT-R* | TCCTCATACTCATCGCTCAA |  |
| *PvRXLR24-qRT-F* | ACCCGCAATTACCGAAAT | qRT-PCR |
| *PvRXLR24-qRT-R* | TACATCAGTACCGCCTCT |  |
| *PvRXLR25-qRT-F* | GAGCAAGAGCCAAGTAGT | qRT-PCR |
| *PvRXLR25-qRT-R* | ACATCGTGTTGACTCCAT |  |
| *PvRXLR27-qRT-F* | AAGTCCAGTATCCACATT | qRT-PCR |
| *PvRXLR27-qRT-R* | AGAGCCATTGAAGTTGAA |  |
| *PvRXLR28-qRT-F* | GTAACATTCCGAGCACTT | qRT-PCR |
| *PvRXLR28-qRT-R* | GTTCCTTATCTTCGTCCAC |  |
| *PvRXLR30-qRT-F* | CGTATATGACATGATGAGG | qRT-PCR |
| *PvRXLR30-qRT-R* | ATATTGTAGGAGCGAAGT |  |
| *PvRXLR49-qRT-F* | GAATTGATTCTTCAGAGATAG | qRT-PCR |
| *PvRXLR49-qRT-R* | CCGCTAGAGATATAATGG |  |
| *PvRXLR54-qRT-F* | ACACAAGGTCGTTGAGAG | qRT-PCR |
| *PvRXLR54-qRT-R* | GTCCGTGAGCAAGAAGAG |  |
| *PvRXLR55-qRT-F* | TATGATGTCGTTTGTAGGT | qRT-PCR |
| *PvRXLR55-qRT-R* | TTTCTTGAATGCGATGAC |  |

| Primer name | | | | | | Primer sequence | | Purpose or vector | | | | | |
| --- | --- | --- | --- | --- | --- | --- | --- | --- | --- | --- | --- | --- | --- |
| *PvRXLR61-qRT-F* | | | | | | GAGCGTATGTTTCTATTAC | | qRT-PCR | | | | | |
| *PvRXLR61-qRT-R* | | | | | | GATGTTCGTTTAGTTGAC | |  |  |  |  |  |  |
| *PvRXLR63-qRT-F* | | | | | | TGTTCGGGTGTTTCAAGA | | qRT-PCR | | | | | |
| *PvRXLR63-qRT-R* | | | | | | AATTGACTCTCCACTCGG | |  |  |  |  |  |  |
| *PvRXLR64-qRT-F* | | | | | | GGCTAATAGAGAACTACGA | | qRT-PCR | | | | | |
| *PvRXLR64-qRT-R* | | | | | | AGGGAATGGTAAACAGAG | |  |  |  |  |  |  |
| *PvRXLR66-qRT-F* | | | | | | AGAGGTGACTACAACAAC | | qRT-PCR | | | | | |
| *PvRXLR66-qRT-R* | | | | | | GTGGACTGCTATATCGTAA | |  |  |  |  |  |  |
| *PvRXLR67-qRT-F* | | | | | | CATCACATACCACGAAGTC | | qRT-PCR | | | | | |
| *PvRXLR67-qRT-R* | | | | | | CTGAGCATTGAGCGAATC | |  |  |  |  |  |  |
| *PvRXLR68-qRT-F* | | | | | | ATCAGAGACTCATCTACAG | | qRT-PCR | | | | | |
| *PvRXLR68-qRT-R* | | | | | | GAAGAAATTGGCTTGGTA | |  |  |  |  |  |  |
| *PvActin-qRT-F* | | | | | | CGATCTCGTATCTGAATA | | qRT-PCR | | | | | |
| *PvActin-qRT-R* | | | | | | CTACATCATCTCATCCAT | |  | | | | | |
| *PvRXLR1-attB1-F* | | AAAAAGCAGGCTCTATGCTGGGAGAC | | | | | | | | pH7FWG2 | | | |
| *PvRXLR1-attB2-R* | | AGAAAGCTGGGTCATGACGCGT | | | | | | | |  |  |  |  |
| *PvRXLR2-attB1-F* | | AAAAAGCAGGCTCTATGCTGGAGATTGCA | | | | | | | | pH7FWG2 | | | |
| *PvRXLR2-attB2-R* | | AGAAAGCTGGGTCAGGCCCCCTTCTCCC | | | | | | | |  |  |  |  |
| *PvRXLR5-attB1-F* | | AAAAAGCAGGCTCTATGATTGACCAA | | | | | | | | pH7FWG2 | | | |
| *PvRXLR5-attB2-R* | | AGAAAGCTGGGTCATGTGTAAC | | | | | | | |  |  |  |  |
| *PvRXLR9-attB1-F* | | AAAAAGCAGGCTCTATGAGCAGCTCTGCG | | | | | | | | pH7FWG2 | | | |
| *PvRXLR9-attB2-R* | | AGAAAGCTGGGTCCCTGCGTTGGTAAGG | | | | | | | |  |  |  |  |
| *PvRXLR10-attB1-F* | | AAAAAGCAGGCTCTATGCGTCGCACTGCT | | | | | | | | pH7FWG2 | | | |
| *PvRXLR10-attB2-R* | | AGAAAGCTGGGTCTTGATTTCTTTTTCTT | | | | | | | |  |  |  |  |
| *PvRXLR11-attB1-F* | | AAAAAGCAGGCTCTATGACGAACGCC | | | | | | | | pH7FWG2 | | | |
| *PvRXLR11-attB2-R* | | AGAAAGCTGGGTCATGAGCGAC | | | | | | | |  |  |  |  |
| *PvRXLR16-attB1-F* | | AAAAAGCAGGCTCTATGACAGGAAATGC | | | | | | | | pH7FWG2 | | | |
| *PvRXLR16-attB2-R* | | AGAAAGCTGGGTCCCTATTGAATAAAG | | | | | | | |  |  |  |  |
| *PvRXLR17-attB1-F* | | AAAAAGCAGGCTCTATGTCGGATAAGCCG | | | | | | | | pH7FWG2 | | | |
| *PvRXLR17-attB2-R* | | AGAAAGCTGGGTCATATTCCTTAACGCA | | | | | | | |  |  |  |  |
| *PvRXLR19-attB1-F* | | AAAAAGCAGGCTCTATGGTAGCCGAC | | | | | | | | pH7FWG2 | | | |
| *PvRXLR19-attB2-R* | | AGAAAGCTGGGTCCCCTTGAAGTGGCCA | | | | | | | |  |  |  |  |
| *PvRXLR22-attB1-F* | | AAAAAGCAGGCTCTATGAATGAAGTTGAA | | | | | | | | pH7FWG2 | | | |
| *PvRXLR22-attB2-R* | | AGAAAGCTGGGTCATCTTGCCGTTCCGC | | | | | | | |  |  |  |  |
| *PvRXLR25-attB1-F* | | | AAAAAGCAGGCTCTATGGTTAACCGAGCCA | | | | | | pH7FWG2 | | | | |
| *PvRXLR25-attB2-R* | | | AGAAAGCTGGGTCCAACTTCCCGTAAAC | | | | | |  |  |  |  |  |
| *PvRXLR27-attB1-F* | | | AAAAAGCAGGCTCTATGGCCGACGAAGTCAAC | | | | | | pH7FWG2 | | | | |
| *PvRXLR27- attB2-R* | | | AGAAAGCTGGGTCGAAGATTATTAGGAG | | | | | |  |  |  |  |  |
| *PvRXLR28-attB1-F* | | | AAAAAGCAGGCTCTATGGCTTGGGGA | | | | | | pH7FWG2 | | | | |
| *PvRXLR28-attB2-R* | | | AGAAAGCTGGGTCCTCGAAGAAT | | | | | |  |  |  |  |  |
| *PvRXLR30-attB1-F* | | | | AAAAAGCAGGCTCTATGGTTGCGACT | | | | | | | pH7FWG2 | | |
| *PvRXLR30-attB2-R* | | | | AGAAAGCTGGGTCTTTGGCCAC | | | | | | |  |  |  |
| Primer name | | | | | | Primer sequence | | Purpose or vector | | | | | |
| *PvRXLR49-attB1-F* | | | | AAAAAGCAGGCTCTATGCTGGATGATGCC | | | | | | | pH7FWG2  pH7FWG2 | | |
| *PvRXLR49-attB2-R* | | | | AGAAAGCTGGGTCAACGCTTCGGAGTCG | | | | | | |  |  |  |
| *PvRXLR54-attB1-F* | | | | AAAAAGCAGGCTCTATGCACGTCTTC | | | | | | |  |  |  |
| *PvRXLR54-attB2-R* | | | | AGAAAGCTGGGTCAGCAGTGCG | | | | | | |  |  |  |
| *PvRXLR55-attB1-F* | | | | AAAAAGCAGGCTCTATGTATGGCATC | | | | | | | pH7FWG2 | | |
| *PvRXLR55-attB2-R* | | | | AGAAAGCTGGGTCCTTGTTCAT | | | | | | |  |  |  |
| *PvRXLR61-attB1-F* | | | | AAAAAGCAGGCTCTATGTATCCCTCACAAATT | | | | | | | pH7FWG2 | | |
| *PvRXLR61-attB2-R* | | | | AGAAAGCTGGGTCATGGCAGCTTGGTAT | | | | | | |  |  |  |
| *PvRXLR63-attB1-F* | | | | AAAAAGCAGGCTCTATGCACCATATAACT | | | | | | | pH7FWG2 | | |
| *PvRXLR63-attB2-R* | | | | AGAAAGCTGGGTCCTTCCAGCATCGCCA | | | | | | |  |  |  |
| *PvRXLR64-attB1-F* | | | | AAAAAGCAGGCTCTATGCAACCAAGCGGA | | | | | | | pH7FWG2 | | |
| *PvRXLR64-attB2-R* | | | | AGAAAGCTGGGTCCATCCAAAAGCTAAA | | | | | | |  |  |  |
| *PvRXLR66-attB1-F* | | | | AAAAAGCAGGCTCTATGCTGACGAGCCCA | | | | | | | pH7FWG2 | | |
| *PvRXLR66-attB2-R* | | | | AGAAAGCTGGGTCATTTTCGGTAAAGTG | | | | | | |  |  |  |
| *PvRXLR67-attB1-F* | | | | AAAAAGCAGGCTCTATGAGCAGTGGTCC | | | | | | | pH7FWG2 | | |
| *PvRXLR67-attB2-R* | | | | AGAAAGCTGGGTCACAGAATGCGGCGC | | | | | | |  |  |  |
| *PvRXLR68-attB1-F* | | | | AAAAAGCAGGCTCTATGGCCCCCTTCTCT | | | | | | | pH7FWG2 | | |
| *PvRXLR68-attB2-R* | | | | AGAAAGCTGGGTCCGAAGCCGATAGTAC | | | | | | |  | | |
| *Nb-EF1α-F* | | | | | | AGAGGCCCTCAGACAAAC | | | | | | qRT-PCR | |
| *Nb-EF1α-R* | | | | | | TAGGTCCAAAGGTCACAA | | | | | |  |  |
| *Nb-PR1b-F* | | | | | | GTGGACACTATACTCAGGTG | | | | | | qRT-PCR | |
| *Nb-PR1b-R* | | | | | | TCCAACTTGGAATCAAAGGG | | | | | |  |  |
| *Nb-PR2b-F* | | | | | | AGGTGTTTGCTATGGAATGC | | | | | | qRT-PCR | |
| *Nb-PR2b-R* | | | | | | TCTGTACCCACCATCTTGC | | | | | |  |  |
| *Nb-ERF1-F* | | | | | | GCTCTTAACGTCGGATGGTC | | | | | | qRT-PCR | |
| *Nb-ERF1-R* | | | | | | AGCCAAACCC TAGCTCCATT | | | | | |  |  |
| *Nb-LOX-F* | | | | | | AAAACCTATGCCTCAAGAAC | | | | | | qRT-PCR | |
| *Nb-LOX-R* | | | | | | ACTGCTGCATAGGCTTTGG | | | | | |  |  |
| *Nb-RbohA-F* | | | | | | GACTCGTTCCAGCGCTCATA | | | | | | qRT-PCR | |
| *Nb-RbohA-R* | | | | | | TGTGCGAAATCGGAACGGTA | | | | | |  |  |
| *Nb-RbohB-F* | | TCACAAGAGCTCAGGCGTTT | | | | | | | | | | qRT-PCR | |
| *Nb-RbohB-R* | | TCATCGAACCGCTTCTCGAC | | | | | | | | | |  |  |
| *PvRxLR28-XhoI-F* | | | | | CCGctcgagATGGCTTGGGGATG | | | | | | | pER8-3xFlag | |
| *PvRxLR28- BstBI-R* | | | | | GGGttcgaaCTCGAAGAATTGGT | | | | | | |  |  |
| *PvRxLR28-NES-XmaI-F* | | TCCCcccgggATGGGTCTGATTGGG | | | | | | | | pGR107 | | | |
| *PvRxLR28-NES-SalI-R* | | ACGCgtcgacTCACTATTTGTTAATATCTAGACCAGC  CAGCTTCAGGGCCAGTTCGTTTCACTTATCGTCATC | | | | | | | |  |  |  |  |
| *PvRxLR28-nes-XmaI-F* | | TCCCcccgggATGGGTCTGATTGGG | | | | | | | | pGR107 | | | |
| *PvRxLR28-nes-SalI-R* | | ACGCgtcgacCTTGTTAGCATCTGCTCCAGCTGCCTTA  AGAGCAAGCTCGTTCTCGAAGAATTGGTAACC | | | | | | | |  |  |  |  |
| *PvRxLR28-NES-attB1* | | AAAAAGCAGGCTCTATGGGTCTGATTGGG | | | | | | | | pH7FWG2 | | | |
| *PvRxLR28-NES-attB2* | | AGAAAGCTGGGTCCTATTTGTTAATATCTAG | | | | | | | |  |  |  |  |
| *PvRxLR28-nes-attB1* | | AAAAAGCAGGCTCTATGGGTCTGATTGGG | | | | | | | | pH7FWG2 | | | |
| Primer name | Primer sequence | | | | | | Purpose or vector | | | | | |  |
| *PvRxLR28-nes-attB2* | | AGAAAGCTGGGTCCTTGTTAGCATCTGCTCC | | | | | | | | pGR107 | | | |
| *PvRxLR28-28-218-XmaI-F* | | TCCCcccgggATGGGTCTGATTGGGCAA | | | | | | | |  |  |  |  |
| *PvRxLR28-28-218-SalI-R* | | ACGCgtcgacTCACTCGAAGAATTGGTA | | | | | | | | pGR107 | | | |
| *PvRxLR28-63-218-XmaI-F* | | TCCCcccgggATGCCTGCCGACAGTCCG | | | | | | | |  |  |  |  |
| *PvRxLR28-63-218-SalI-R* | | ACGCgtcgacTCACTCGAAGAATTGGTA | | | | | | | | pGR107 | | | |
| *PvRxLR28-93-218-XmaI-F* | | TCCCcccgggATGAGCAGTAACCAGCCC | | | | | | | |  |  |  |  |
| *PvRxLR28-93-218-SalI-R* | | ACGCgtcgacTCACTCGAAGAATTGGTA | | | | | | | | pGR107 | | | |
| *PvRxLR28-133-218-XmaI-F* | | TCCCcccgggATGCCGATCACCGGACTC | | | | | | | |  |  |  |  |
| *PvRxLR28-133-218-SalI-R* | | ACGCgtcgacTCACTCGAAGAATTGGTA | | | | | | | | pGR107 | | | |
| *PvRxLR28-133-200-XmaI-F* | | TCCCcccgggATGCCGATCACCGGACTC | | | | | | | |  |  |  |  |
| *PvRxLR28-133-200-SalI-R* | ACGCgtcgacTCACGCCACCAAATTTTC | | | | | | pGR107 | | | | | |  |
| *PvRxLR28-150-200-XmaI-F* | TCCCcccgggATGGGTGAGCAAATTTCA | | | | | |  |  |  |  |  |  |  |
| *PvRxLR28-150-200-SalI-R* | ACGCgtcgacTCACGCCACCAAATTTTC | | | | | | pGR107 | | | | | |  |
| *PvRxLR28-150-180-XmaI-F* | TCCCcccgggATGGGTGAGCAAATTTCA | | | | | |  |  |  |  |  |  |  |
| *PvRxLR28-150-180-SalI-R* | ACGCgtcgacTCATGTCGGGGGTCCAGA | | | | | |  | | | | | |  |
|  |  | | | | | |  |  |  |  |  |  |  |
|  |  | | | | | |  | | | | | |  |
|  |  | | | | | |  |  |  |  |  |  |  |
|  |  | | | | | |  | | | | | |  |
|  |  | | | | | |  |  |  |  |  |  |  |
|  |  | | | | | |  | | | | | |  |
|  |  | | | | | |  |  |  |  |  |  |  |
|  |  | | | | | |  | | | | | |  |
|  |  | | | | | |  |  |  |  |  |  |  |
|  |  | | | | | |  | | | | | |  |
|  |  | | | | | |  |  |  |  |  |  |  |
|  |  | | | | | |  | | | | | |  |
|  |  | | | | | |  |  |  |  |  |  |  |
|  |  | | | | | |  | | | | | |  |
|  |  | | | | | |  |  |  |  |  |  |  |
|  |  | | | | | |  | | | | | |  |
|  |  | | | | | |  |  |  |  |  |  |  |
|  |  | | | | | |  | | | | | |  |
|  |  | | | | | |  |  |  |  |  |  |  |
|  |  | | | | | |  | | | | | |  |
|  |  | | | | | |  |  |  |  |  |  |  |
|  |  | | | | | |  | | | | | |  |
|  |  | | | | | |  |  |  |  |  |  |  |
|  |  | | | | | |  | | | | | |  |
|  |  | | | | | |  |  |  |  |  |  |  |
|  |  | | | | | |  | | | | | |  |
|  |  | | | | | |  | | | | | |  |

Primers were designed based on the results of previous study (Yin et al., 2015).
