# Supplementary figures and images for: Studying the Mechanism of Plasmopara viticola RxLR Effectors on Suppressing Plant Immunity
Source: Front Microbiol. 2016 May 18;7:709. doi: 10.3389/fmicb.2016.00709 (PMC4870276; doi:10.3389/fmicb.2016.00709)

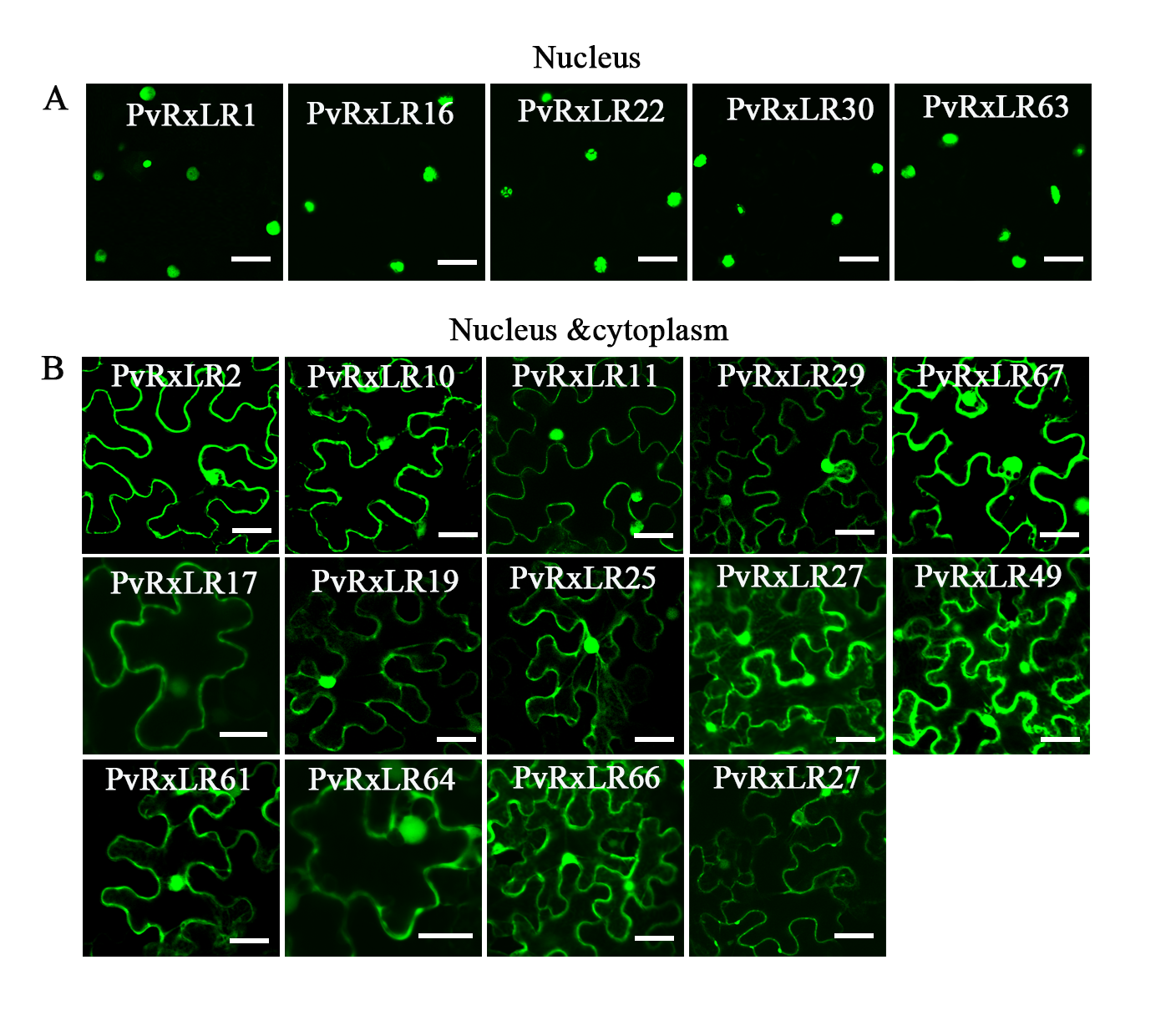

Supplement: Figure S1 — Subcellular localizations of PvRxLRs in N. benthamiana leaf tissues. [file Image1.TIF]

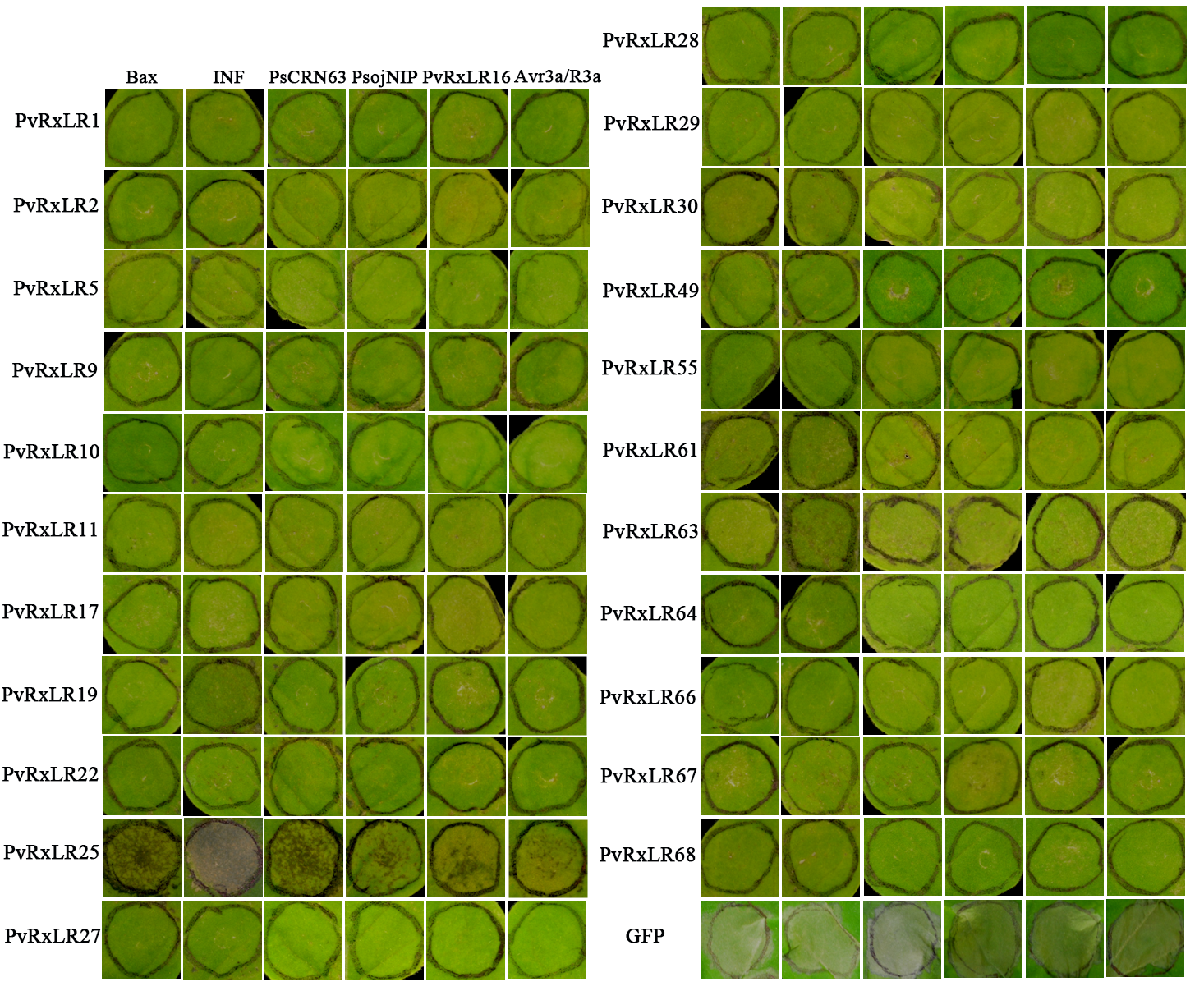

Supplement: Figure S2 — Suppression of different elicitors-triggered cell death in N. benthamiana by PvRxLR effectors. [file Image2.TIF]
